# Treatises of Fistula in Ano, Hæmorrhoids and Clysters

**Published:** 1911-09

**Authors:** 


**Treatises of Fistula in Ano, Hæmorrhoids and Clysters.** By JOHN ARDERNE. From an Early Fifteenth-Century Manuscript Translation. Edited by D'ARCY POWER. London: Published for the Early English Text Society by Kegan Paul, Trench, Trübner & Co. Ltd. 1910.

The temptation to deal at length with this admirable volume is a strong one, but must be resisted on account of want of space. Our readers must be referred to the book itself for a knowledge of the originality and courage of this fourteenth-century surgeon. But not only will the practical surgeon be interested in its study, for there is much to attract the student of the history of medicine, the folklorist, and the philologist.

Arderne's work has been most excellently presented by Mr. D'Arcy Power in a scholarly introduction and with very full notes upon points requiring elucidation. Our thanks are cordially given to him for his careful editing, and to the Early English Text Society for adding this volume to the not inconsiderable number of publications which it has previously issued bearing on various aspects of our professional work.

Local readers may be reminded that the Bristol Public Library contains a fifteenth-century illustrated MS. of the surgical work of Guy de Chauliac, a contemporary of John Arderne.

